# Hydroxynitrile lyases from cyanogenic millipedes: molecular cloning, heterologous expression, and whole-cell biocatalysis for the production of (*R*)-mandelonitrile

**DOI:** 10.1038/s41598-018-20190-x

**Published:** 2018-02-14

**Authors:** Takuya Yamaguchi, Aem Nuylert, Atsutoshi Ina, Tsutomu Tanabe, Yasuhisa Asano

**Affiliations:** 1 Biotechnology Research Center and Department of Biotechnology, Toyama Prefectural University, 5180 Kurokawa, Imizu, Toyama, 939–0398 Japan; 2 Asano Active Enzyme Molecule Project, ERATO, JST, 5180 Kurokawa, Imizu, Toyama, 939–0398 Japan; 3 Faculty of Education, Kumamoto University, Kumamoto, 860–8555 Japan

## Abstract

Hydroxynitrile lyases (HNLs), which are key enzymes in cyanogenesis, catalyze the cleavage of cyanohydrins into carbonyl compounds and hydrogen cyanide. Since HNLs also catalyze the reverse reaction, they are used industrially for the asymmetric synthesis of cyanohydrins, which are valuable building blocks of pharmaceuticals and fine chemicals. HNLs have been isolated from cyanogenic plants and bacteria. Recently, an HNL from the cyanogenic millipede *Chamberlinius hualienensis* was shown to have the highest specific activity for (*R*)-mandelonitrile synthesis, along with high stability and enantioselectivity. However, no HNLs have been isolated from other cyanogenic millipedes. We identified and characterized HNLs from 10 cyanogenic millipedes in the Paradoxosomatidae and Xystodesmidae. Sequence analyses showed that HNLs are conserved among cyanogenic millipedes and likely evolved from one ancestral gene. The HNL from *Parafontaria tonominea* was expressed in *Escherichia coli* SHuffle T7 and showed high specific activity for (*R*)-mandelonitrile synthesis and stability at a range of pHs and temperatures. The stability of millipede HNLs is likely due to disulfide bond(s). The *E. coli* cells expressing HNL produced (*R*)-mandelonitrile with 97.6% enantiomeric excess without organic solvents. These results demonstrate that cyanogenic millipedes are a valuable source of HNLs with high specific activity and stability.

## Introduction

Hydroxynitrile lyases (HNLs; EC 4.1.2.X, X = 10, 11, 46, and 47) catalyze the reversible conversion of cyanohydrins into carbonyl compounds and hydrogen cyanide. In cyanogenic organisms, HNLs play a crucial role in releasing hydrogen cyanide as a defensive agent^1^. In industry, HNLs have been used to synthesize chiral cyanohydrins^2^, which are useful building blocks for the synthesis of pharmaceuticals, fine chemicals, and agrochemicals^3^. To date, most HNLs have been identified from cyanogenic plants^4,5^, although several bacteria also contain HNLs^6–8^. The structures of HNLs show similarities to those of α/β-hydrolases, oxidoreductases, carboxypeptidases, Zn^2+^-dependent alcohol dehydrogenases, and Mn^2+^-dependent cupins^8–12^. The HNL (PaHNL) from almond (*Prunus amygdalus* Batsch) shows high specific activity for the synthesis of (*R*)-mandelonitrile and strong stability; therefore, it is a valuable catalyst in industry^13^.

Millipedes are a highly diverged group, and more than 12000 species have been described worldwide^14^. Among them, polydesmid millipedes are generally cyanogenic^15^. When cyanogenic millipedes are alarmed, (*R*)-mandelonitrile stored in the reservoir of defensive glands is degraded into benzaldehyde and hydrogen cyanide. Both compounds are secreted externally through ozopores on some segments of the paired paranota^16^. Recently, an HNL (ChuaHNL) from the invasive swarm-forming polydesmid millipede, *Chamberlinius hualienensis* Wang, was isolated and characterized^17,18^. ChuaHNL showed not only the highest specific activity for (*R*)-mandelonitrile synthesis among all characterized HNLs, but also high enantioselectivity and stability at a range of temperatures and pHs. Other cyanogenic millipedes may also contain HNLs with high specific activity and stability. However, no other millipede HNLs have been identified because the genome sequences of cyanogenic millipedes are not available and ChuaHNL does not show similarity to any other proteins^18^.

In this study, we purified an HNL from a cyanogenic millipede and determined its cDNA sequence. Next, we designed degenerate primers to amplify cDNAs encoding HNL from cyanogenic millipedes belonging to the Paradoxosomatidae and Xystodesmidae families. All of these eleven cDNAs encoding HNL were heterologously expressed in insect cells, five cDNAs encoding HNL were able to express in *Escherichia coli*, and transgenic *E. coli* cells expressing a cDNA encoding HNL were used as whole-cell catalysts to synthesize (*R*)-mandelonitrile.

## Results

### Purification of HNL (NttHNL) from *Nedyopus tambanus tambanus*

**Figure 1:**
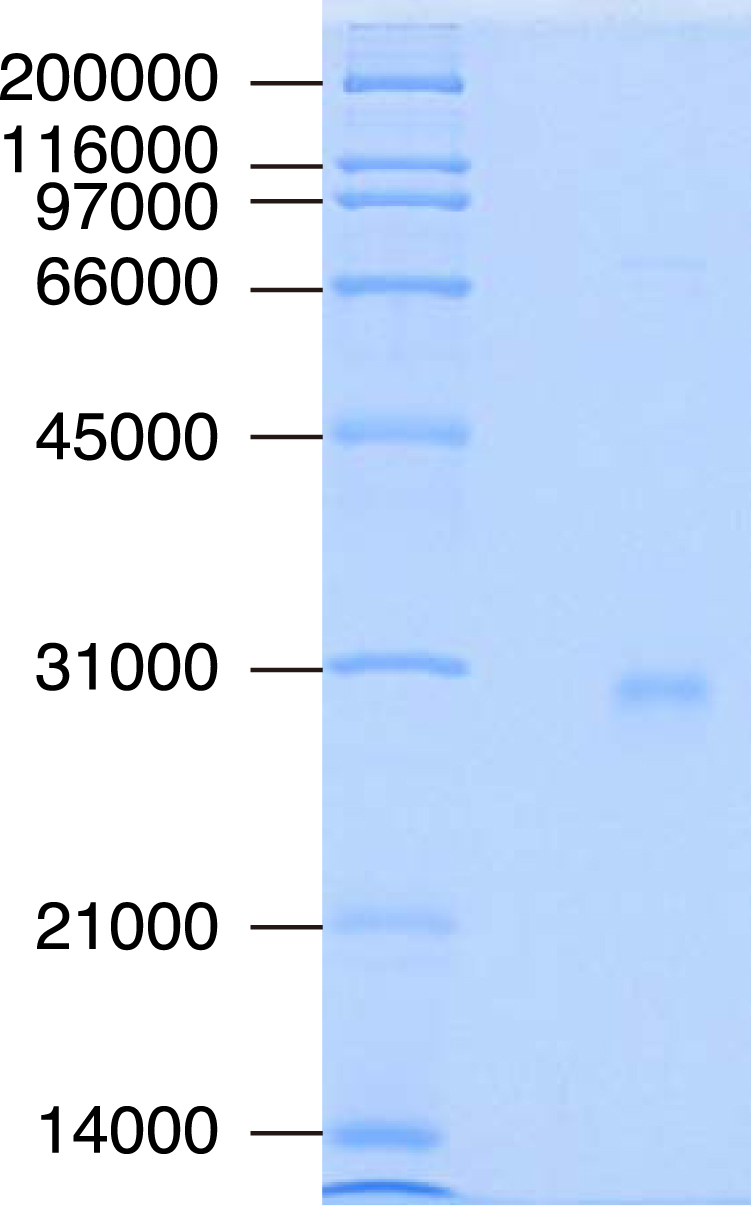
SDS-PAGE analysis of HNL purified from *N. tambanus tambanus*. HNL purified from *N. tambanus tambanus* was separated by 12% SDS-PAGE with Broad Range (Bio-Rad Laboratories) molecular weight markers. The full-length gel is presented in Fig. S4.

We collected *Nedyopus tambanus tambanus*, which swarms every April at our facility, and extracted and purified NttHNL (Fig. S1). The crude extract from this millipede showed specific activity of 65.9 U/mg in the (*R*)-mandelonitrile synthetic reaction. Then, NttHNL was purified 38.6-fold from the extract with 20.2% yield. When the purified enzyme was separated by SDS-PAGE, it showed a single band with a molecular weight of 28800 Da (Fig. 1). The relative molecular weight of the native enzyme was determined to be 52400 Da by gel filtration, indicating that this enzyme functions as a dimer, like ChuaHNL^18^. NttHNL was *R*-selective HNL and the specific activity of purified NttHNL for (*R*)-mandelonitrile synthesis was 4702 U/mg, lower than that of ChuaHNL (7420 U/mg)^18^, but three times higher than that of PaHNL (1450 U/mg)^19^, which is used industrially. These results suggested that cyanogenic millipedes are a good source of HNLs with high specific activity for cyanohydrin synthesis.

### Molecular cloning of cDNAs encoding HNLs from cyanogenic millipedes

**Figure 2:**
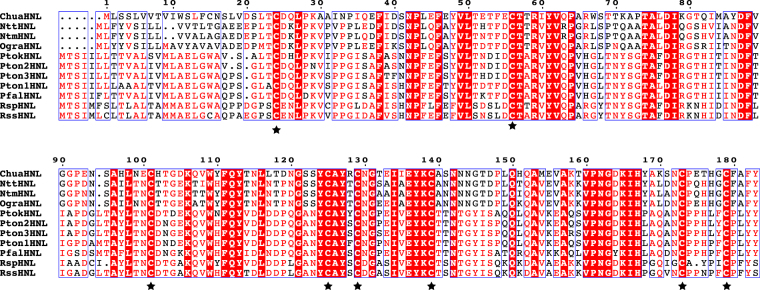
Amino acid sequence alignment of HNLs from millipedes. Multiple sequence alignment was visualized by ESPript 3 (http://espript.ibcp.fr/ESPript/ESPript/). Red background shows strictly conserved residues; red letters indicate residues well conserved within a group according to a Raisler matrix; and remainder are shown in black. Residues conserved between groups are boxed. Stars indicate Cys residues conserved among millipede HNLs.

Based on the protein sequence of NttHNL, the cDNA encoding NttHNL was cloned after 5′- and 3′-rapid amplification of cDNA ends (RACE). The deduced amino acid sequence of NttHNL was 64% identical to that of ChuaHNL, suggesting that HNLs are conserved among cyanogenic millipedes. To clone cDNAs encoding HNLs from cyanogenic millipedes collected from various locations in Japan (Table 1), degenerate primers were designed on the basis of homologous sequences between NttHNL and ChuaHNL. The degenerate primers were used to amplify partial sequences of cDNAs encoding HNLs from *Nedyopus tambanus mangaesinus* (NtmHNL), *Oxidus gracilis* (OgraHNL), and *Parafontaria tonominea* species complex 1 (Pton1HNL). The degenerate primers designed from the conserved sequence of the above-mentioned HNLs also amplified partial sequences of cDNAs encoding HNLs from *P. falcifera* (PfalHNL), *P. tonominea* species complex 2 and 3 (Pton2HNL and Pton3HNL, respectively), *P. tokaiensis* (PtokHNL), *Riukiaria semicircularis semicircularis* (RssHNL), and *Riukiaria* sp. (RspHNL). Full-length cDNAs encoding these HNLs were obtained by 5′- and 3′-RACE. Deduced amino sequences of these HNLs showed 45–66% similarities to ChuaHNL at the amino acid level, but showed no similarity to plant and bacterial HNLs. All of the newly identified HNLs from millipedes were predicted to contain glycosylation sites and secretion signal sequences (Table S1), consistent with the fact that ChuaHNL is a glycosylated protein and that HNLs are secreted into reaction chambers of defensive glands^18^. Amino acid sequence analyses showed that eight Cys residues were conserved among all the millipede HNLs (Fig. 2). Considering that iodoacetic acid inhibits ChuaHNL activity^18^, Cys residues are likely to be crucial residues in millipede HNLs.

**Table 1:** Millipedes and their collection sites.

Species	Collection site
*Nedyopus tambanus tambanus* (Attems)	36°42′25.4“N 137°05′51.3“E
*Nedyopus tambanus mangaesinus* (Attems)	36°39′38.4“N 137°06′10.0“E
*Oxidus gracilis* (C. L. Koch)	36°42′29.9“N 137°05′54.7“E
*Parafontaria falcifera* (Verhoeff)	36°41′38.9“N 137°08′55.5“E
*Parafontaria tokaiensis* Tanabe	34°50′44.8“N 137°53′59.0“E
*Parafontaria tonominea* species complex 1	35°35′29.8“N 136°56′44.0“E
*Parafontaria tonominea* species complex 2	34°47′55.0“N 138°04′29.4“E
*Parafontaria tonominea* species complex 3	34°27′58.8“N 135°52′39.5“E
*Riukiaria semicircularis semicircularis* (Takakuwa)	32°30′53.7“N 130°44′39.2“E
*Riukiaria* sp.	31°20′22.2“N 130°27′09.7“E

**Figure 3:**
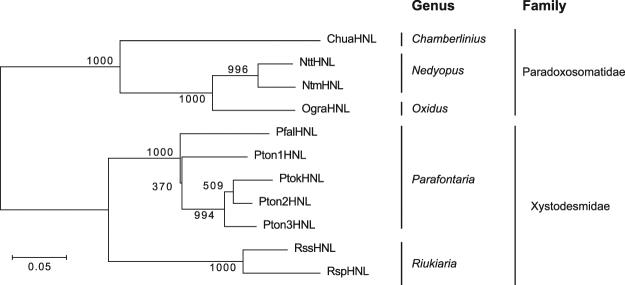
Phylogenetic analysis of HNLs from cyanogenic millipedes. Phylogenetic tree was constructed by the neighbor-joining method with 1000 bootstrap replicates. Bar indicates 5% divergence.

In the phylogenetic analysis, the HNLs were separated into two branches (Fig. 3) that corresponded to the two families (Xystodesmidae and Paradoxosomatidae). The HNLs from *Nedyopus*, *Parafontaria*, and *Riukiaria* clustered together (Fig. 3). These results suggested that genes encoding HNL likely evolved from one ancestral gene during the evolution of polydesmoid millipedes.

### Recombinant production of millipede HNLs in heterologous hosts

To characterize the HNLs from millipedes, recombinant HNLs were produced in heterologous expression hosts. First, we chose the baculovirus–insect cell expression system, because it has the highest similarity to millipedes in terms of the patterns and capacity of posttranslational modifications. In this system, the millipede HNLs were secreted into the medium and all of them catalyzed the asymmetric synthesis of (*R*)-mandelonitrile. Their concentrations ranged from 0.4 to 10.5 U/mL culture medium (Table 2). These results confirmed that the cloned cDNAs encoded functionally active HNLs.

**Table 2:** Heterologous expression of *HNL* genes in insect cells and *E. coli*.

	Sf9	*E. coli* BL21(DE3)	*E. coli* SHuffle T7	
	Total activity (U/mL culture)	Total activity (U/mL culture)	Total activity (U/mL culture)	Specific activity^a^ (U/mg protein)
ChuaHNL	0.4 ± 0.1	ND^b^	ND	NT^c^
NttHNL	1.7 ± 0.1	ND	1.6 ± 0.3	1945 ± 270
NtmHNL	3.5 ± 0.3	ND	10.6 ± 3.6	1997 ± 178
OgraHNL	5.3 ± 0.3	ND	2.2 ± 0.3	2741 ± 149
PfalHNL	2.2 ± 0.1	ND	ND	NT
PtokHNL	10.5 ± 0.7	ND	ND	NT
Pton1HNL	3.6 ± 0.04	ND	ND	NT
Pton2HNL	9.9 ± 0.5	ND	16.9 ± 2.1	3371 ± 208
Pton3HNL	9.7 ± 0.3	ND	40.3 ± 6.2	2140 ± 162
RssHNL	1.7 ± 0.3	ND	ND	NT
RspHNL	3.5 ± 0.6	ND	ND	NT

^a^Specific activity was determined using purified enzymes, ^b^ND; Not detected, ^c^NT; Not tested.

Next, we tried to express the millipede HNLs in *E. coli*, which is easily transformed and can be cultured in a short time for large-scale production. However, the millipede HNLs were not expressed in *E. coli* BL21(DE3) (Table 2), which is generally used for heterologous protein production. The millipede HNLs were found to contain conserved eight Cys residues (Fig. 2), which might be involved in the formation of disulfide bonds. Proteins that require disulfide bonds for their folding and stability have been shown to be poorly expressed, misfolded, or inactive when expressed in the cytoplasm of wild-type *E. coli* strains^20^. Therefore, we used the genetically engineered strain *E. coli* SHuffle T7 as the expression host. This strain constitutively expresses disulfide bond isomerase DsbC, which corrects mis-oxidized proteins^20,21^. In SHuffle T7, we detected the expression of NttHNL, NtmHNL, OgraHNL, Pton2HNL, and Pton3HNL. Among them, the HNL showing the highest concentration was Pton3HNL (40.3 U/mL culture medium), higher than the highest concentration in the insect cell expression system (Table 2). The specific activities of purified recombinant NttHNL, NtmHNL, OgraHNL, Pton2HNL, and Pton3HNL were 1945 U/mg, 1997 U/mg, 2741 U/mg, 3371 U/mg, and 2140 U/mg, respectively (Table 2), all of which were higher than that of PaHNL (1450 U/mg). When the same amounts of purified enzymes (10 U/mL) were used in 5 min of reaction, (*R*)-mandelonitrile was obtained with an enantiomeric excess of 85% (NttHNL), 88% (NtmHNL), 83% (OgraHNL), 91% (Pton2HNL), and 91% (Pton3HNL), indicating that recombinant millipede HNLs have excellent stereoselectivity.

### Effect of temperature and pH on enzyme activity and stability of recombinant Pton3HNL

**Figure 4:**
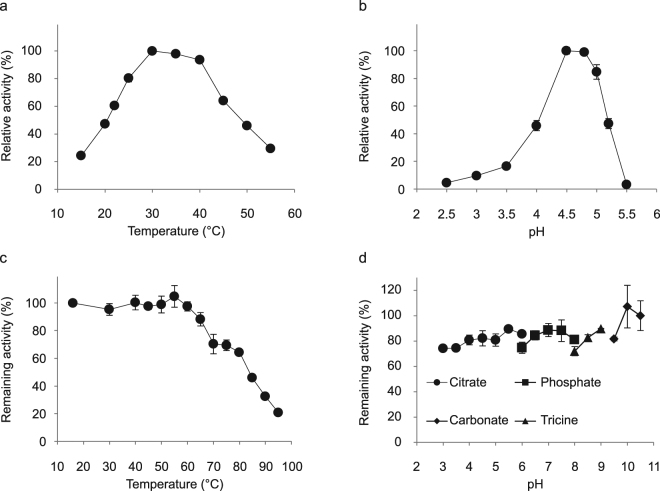
Effect of temperature and pH on recombinant Pton3HNL. (**a**) Optimum temperature. Reaction was performed at 15–55 °C for 5 min in 300 mM sodium citrate buffer, pH 4.2. (**b**) Optimum pH. Reaction was performed at 22 °C for 5 min in 300 mM sodium citrate buffer (pH 2.5–5.5). (**c**) Temperature stability. Remaining activity was measured after incubation of Pton3HNL at various temperatures (15–95 °C) in 10 mM potassium phosphate buffer, pH 7.0, for 60 min. (**d**) pH stability. Remaining activity was measured after incubation of Pton3HNL at 25 °C for 60 min with the following buffers: 100 mM sodium citrate buffer (pH 3.0–6.0); 100 mM potassium phosphate buffer (pH 6.0–8.0), 100 mM Tricine-NaOH buffer (pH 8.0–9.0), and 100 mM sodium carbonate buffer (pH 9.5–10.5). Values are mean ± SD; *n* = 3.

Since Pton3HNL showed the highest expression level in *E. coli* SHuffle T7, we used purified recombinant Pton3HNL to evaluate the effects of temperature and pH on enzyme activity and stability. The optimal temperature and pH ranges of the enzyme were 30–40 °C and 4.5–4.8, respectively (Fig. 4a,b). After a 1-h incubation in 10 mM potassium phosphate buffer (KPB; pH 7.0), Pton3HNL retained full activity between 15 °C and 60 °C (Fig. 4c). After a 1-h incubation at 25 °C, Pton3HNL retained activity over the pH range of 3–10.5 (Fig. 4d). The pH and temperature stabilities of the recombinant Pton3HNL were similar or better than those of ChuaHNL purified from millipedes^18^.

Circular dichroism (CD) was utilized as an additional tool to examine the pH and thermal stabilities of the purified recombinant Pton3HNL. The CD spectrum of Pton3HNL exhibited that the enzyme contains α-helix (222 nm) and random structures, based on a decrease in mean residue ellipticity at 200–210 nm (Fig. S2). This result is corresponding to the CD spectrum analysis using DichroWeb, which suggested that the enzyme contains 29%, 30% and 41% for α-helices, β-strands, and random structures, respectively. After a 1-h incubation in the pH range of 3.0–10.0 at 25 °C, CD spectra of Pton3HNL were converged together at the pH ranges of 3.0–9.0 (Fig. S2a). The proportion of β-strands at pH 10.0 was slightly decreased (25%) from those (30%) at pH 7.0. After 1-h incubation in KPB (pH 7.0), Pton3HNL was stable at temperature ≤70 °C and the curves converged together (Fig. S2b). In the temperature ranges of 90–100 °C, the curves were different from those at 25–70 °C, the percentages of β-strands were notably decreased (4% at 90 °C and 5% at 100 °C). The results are in agreement with that the recombinant Pton3HNL almost lost its activity after 1-h incubation at 95 °C (Fig. 4c). Taken together, the recombinant Pton3HNL has excellent stabilities toward pH and temperatures.

### Synthesis of (*R*)-mandelonitrile using whole recombinant *E. coli* cells

**Figure 5:**
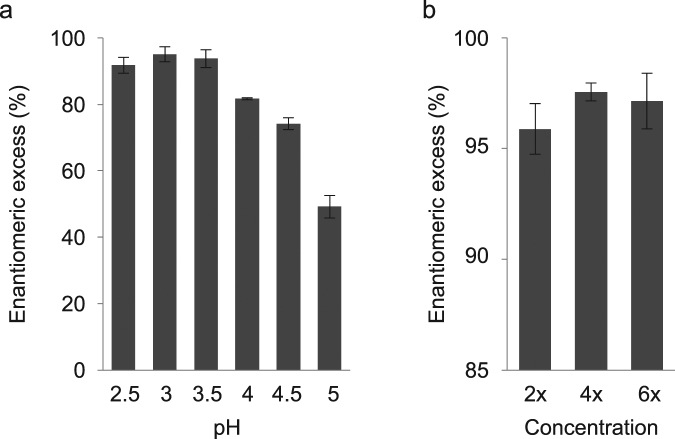
Synthesis of (*R*)-mandelonitrile using *E. coli* cells expressing Pton3HNL in aqueous solution. (**a**) Relationship between pH and enantiomeric excess (ee). *E. coli* cells harvested from 0.4 mL culture were reacted in 0.2 mL 300 mM sodium citrate buffer (pH 2.5–5.0) containing 50 mM benzaldehyde and 100 mM KCN at 22 °C for 5 min. (**b**) Relationship between cell amounts and ee. *E. coli* cells harvested from 0.4 (2×), 0.8 (4×), or 1.2 mL (6×) were reacted in 0.2 mL 0.3 M sodium citrate buffer, pH 3.0, containing 50 mM benzaldehyde and 100 mM KCN at 22 °C for 5 min.

Using whole cells as biocatalysts circumvents cell lysis and enzyme purification steps, and hence, substantially cuts costs^22^. The feasibility of a whole-cell reaction system to produce (*R*)-mandelonitrile was tested using *E. coli* SHuffle T7 cells expressing Pton3HNL. In initial assay conditions (pH 5.0) without organic solvents, *E. coli* cells catalyzed the synthesis of (*R*)-mandelonitrile with 49.2% enantiomeric excess (Fig. 5-a). It has been reported that low pH suppresses the spontaneous chemical reaction producing racemic mandelonitrile^23^. Therefore, we synthesized (*R*)-mandelonitrile in the pH range of 2.5–5.0. The enantiomeric excess of (*R*)-mandelonitrile synthesis reached 95.1% at pH 3.0 (Fig. 5a). Finally, we optimized the amount of recombinant cells to produce a maximum enantiomeric excess of 97.6% (Fig. 5b). A HPLC chromatogram of (*R*)-mandelonitrile synthesis under the optimized condition was shown in Fig. S3. These results indicated that Pton3HNL expressed in SHuffle T7 has the potential for industrial use.

## Discussion

Previous studies have focused on HNLs from plants and bacteria^5,7^, since these HNLs are valuable enzymes for the asymmetric synthesis of cyanohydrins. Recently, an HNL from the cyanogenic millipede *C. hualienensis* was found to have the highest specific activity toward (*R*)-mandelonitrile synthesis detected to date, along with excellent temperature and pH stabilities^18^. The results of the present study showed that HNLs are conserved among cyanogenic millipedes and that recombinant HNLs produced in *E. coli* have high specific activity and excellent temperature and pH stabilities. The results also showed that recombinant *E. coli* cells can synthesize asymmetric (*R*)-mandelonitrile with excellent enantiomeric excess in an aqueous solution.

To use millipede HNLs as biocatalysts, their overexpression in heterologous host is preferable due to the limited availability of millipedes in nature. The baculovirus–insect cell expression system could produce all of eleven millipede HNLs tested as functional proteins. However, growth of cells and cost of medium are inferior to *E. coli* and yeast systems^24^. The methylotrophic yeast *Pichia pastoris* is often utilized as heterologous host of plant HNLs^25–28^. The yeast could produce functional ChuaHNL^18^ yielding 0.9 U/mL culture. The production level was not sufficient. It is known that co-overexpression of genes encoding protein disulfide isomerase and disulfide-rich protein in *P. pastoris* can increase production level of disulfide-rich protein^29,30^. This method may improve expression of millipede HNLs in *P. pastoris*. Although expression of ChuaHNL was not detected in *E. coli*, five other millipede HNLs were successfully expressed in *E. coli* in this study (Table 2). The HNL showing the highest concentration in culture medium was Pton3HNL (40.3 U/mL) and the purified recombinant enzyme showed high specific activity (2140 U/mg) and strong temperature and pH stabilities (Fig. 4), like ChuaHNL. Since synthesis of cyanohydrins is usually carried out at low pH to avoid undesirable non-enzymatic reactions, the excellent stability of millipede HNLs at low pH is an attractive trait for industrial use. Glycosylation is crucial for the stability of HNLs from plants such as *Prunus amygdalus* and *Passiflora edulis* Sims^27,31^. ChuaHNL is a glycoprotein^18^ and the HNLs identified in this study have putative glycosylation sites (Table S1). Although *E. coli* does not produce glycosylated proteins, recombinant Pton3HNL showed high temperature and pH stabilities (Fig. 4c,d). The millipede HNLs were found to contain eight highly conserved Cys residues (Fig. 2). Considering that *E. coli* cells were able to produce functional millipede HNLs in the presence of DsbC, but not in its absence (Table 2), Cys residues likely form disulfide bond(s) that stabilize the protein^32^. Thus, the stability of millipede HNLs is likely due to their disulfide bond(s).

The synthesis of cyanohydrins is usually carried out in aqueous–organic two-phase systems with the enzyme in the aqueous phase^23,33,34^, or in micro-aqueous organic solvents using immobilized enzymes^35^. During these processes, laborious steps such as cell disruption, purification, and/or concentration are required to prepare the enzyme solution. Whole-cell biocatalysis can circumvent these laborious steps and simplify the reaction^36,37^. In this context, we demonstrated that *E. coli* cells expressing Pton3HNL were able to produce (*R*)-mandelonitrile with an enantiomeric excess of 97.6%, without using organic solvents (Fig. 5). Similarly, *E. coli* cells expressing HNL from *Arabidopsis thaliana* were shown to catalyze the synthesis of (*R*)-mandelonitrile with an excellent enantiomeric excess in a monophasic micro-aqueous reaction system^38^. Our whole-cell catalysis utilizing millipede HNLs reduces the need for organic solvents, thereby reducing hazardous waste and environmental pollution^39^.

The arthropod class Diplopoda (millipedes) contains more than 12000 described species^18^ and is one of the most ancient groups of terrestrial animals. Among them, polydesmoid millipedes, which comprise more than 5000 species^14^, generally biosynthesize (*R*)-mandelonitrile from L-phenylalanine^40^ and accumulate this compound as a cyanide source to release hydrogen cyanide as a defensive agent^15^. In this study, we showed that HNLs are conserved among cyanogenic millipedes (Fig. 2) and likely evolved from one ancestral HNL gene during the evolution of polydesmoid millipedes (Fig. 3). Thus, millipedes are a large and diverse source of industrial HNLs with high specific activity and strong temperature and pH stabilities.

## Materials and Methods

### Millipedes

Millipedes were collected from 10 locations in Honshu and Kyushu, Japan (for details see Table 1). *Parafontaria. tonominea* is recognized as a species complex since it represents an aggregation of very closely related forms comprising diverse evolutionary states ranging from geographic variation to full reproductive units^41,42^. In this study, we tentatively assigned species numbers to *P. tonominea* based on their collection sites (Table 1). Millipedes were reared in litter of Japanese cedar (*Cryptomeria japonica* D. Don) at room temperature until use.

### Enzyme assay

The synthesis of mandelonitrile from benzaldehyde was analyzed by HPLC with a chiral column as described in a previous report^18^ with slight modifications. The enzyme samples were mixed with 200 µl 300 mM citrate buffer (pH 4.2) containing 50 mM benzaldehyde and 100 mM potassium cyanide. The mixture was incubated at 22 °C for 5 min. Then, 50 µl reactant was added to 450 µl of *n*-hexane:2-propanol = 85:15, mixed vigorously, and the mixture was centrifuged at 20000 *g* for 3 min. A 5-µl aliquot of the organic phase was analyzed as described previously^40^ using a UFLC Prominense Liquid Chromatograph LC-20AD equipped with a CHIRALCEL OJ-H column (particle size: 5 µm; 4.6 mm i.d. × 250 mm; Daicel Corporation, Tokyo, Japan) connected to a Prominence UV-Vis detector SPD-20A (Shimadzu, Kyoto, Japan). The separation conditions were as follows: mobile phase, *n*-hexane:2-propanol (85:15); flow rate, 1 mL/min. Eluted products were detected by monitoring absorbance at 254 nm.

### Purification of HNL from *N. tambanus tambanus*

NttHNL was extracted from *N. tambanus tambanus* in phosphate buffered saline (PBS; 1.47 mM KH_2_PO_4_, 8.1 mM Na_2_HPO_4_, 137 mM NaCl, 2.7 mM KCl, pH 7.4) and then purified. After adding ammonium sulfate to 30% saturation, the crude extract was loaded onto a TOYOPEARL Butyl-650M column (Tosoh, Tokyo, Japan). The adsorbed proteins were eluted by a step-wise gradient of ammonium sulfate in PBS (20%, 10%, and 0% saturation). The active fractions were loaded onto a PD-10 column (GE Healthcare, Little Chalfont, UK) equilibrated with 10 mM Tris-HCl (pH 9.0). The eluted proteins were loaded onto a MonoQ 5/50 GL column (GE Healthcare), and eluted with a linear gradient of sodium chloride (0–100 mM) in the same buffer. The active fractions were pooled and concentrated using a centrifugal filtration device (Amicon Ultra 0.5 Centrifugal Filter Unit with ultracel-10 membrane; Merck Millipore, Billerica, MA, USA). The concentrated enzyme solution was applied to a Superdex 200 10/300 column (GE Healthcare) equilibrated with PBS.

### Protein sequencing

The N-terminal amino acid sequencing of purified NttHNL was performed at Nippi Inc. (Tokyo, Japan) using a Procise492HT protein sequencer (Applied Biosystems, Foster city, CA, USA). The internal amino acid sequence was determined as described previously^43^. Briefly, after separation of purified NttHNL by 12% SDS-PAGE, the protein was visualized by Coomassie Brilliant Blue staining, excised, and then treated with trypsin (sequencing-grade modified trypsin; Promega, Madison, WI, USA). The digested peptides were analyzed using a nanoflow liquid chromatography–tandem mass spectrometry system. Desalting and separation were performed on a nanoACQUITY UPLC Symmetry C18 trap column (particle size: 5 μm; 180 μm i.d. × 20 mm, Waters, Milford, MA, USA) and an ACUITY UPLC Peptide CSH C18 nanoACQUITY column (particle size: 1.7 μm; 75 μm i.d. × 200 mm, Waters), respectively. The mobile phases were water containing 0.1% formic acid (A) and acetonitrile containing 0.1% formic acid (B). The elution profile was a linear gradient of B (10–80%) at a flow rate of 300 nL/min for 90 min. Mass spectrometry was simultaneously performed in positive electrospray ionization mode with a capillary voltage of 3 kV and cone voltage of 40 V using a SYNAPT G2-Si high-resolution quadrupole TOF MS (Waters). [Glu1]-fibrinopeptide B was used as a mass calibrant. The MS/MS spectra were analyzed using BioLynx (Waters).

### Molecular cloning of cDNAs encoding NttHNL and HNLs from millipedes

Total RNA was prepared from millipedes using TRIzol reagent (Thermo Fisher Scientific, Waltham, MA, USA) and the Direct-zol MiniPrep kit (Zymo Research, Irvine, CA, USA). RACE-ready cDNA was synthesized using the GeneRacer Kit (Thermo Fisher Scientific) and the SMART RACE cDNA amplification kit (Clontech Laboratories, Palo Alto, CA, USA). In cDNA cloning, degenerate oligonucleotide primers were designed on the basis of N-terminal and internal amino sequences of NttHNL or consensus amino acid sequences between ChuaHNL (GenBank: BAS02094) and NttHNL. The PCRs were performed using Tks Gflex DNA polymerase (Takara, Shiga, Japan) as the Taq DNA polymerase, and the PCR products were cloned into the pCR-Blunt vector (Thermo Fisher Scientific) and sequenced. Primers for 5′-and 3′-RACE were designed from partial sequences of cDNAs encoding HNLs. The RACE products were cloned into the pCR-Blunt vector and sequenced. Finally, the coding sequences of HNLs were amplified and cloned into the pCR-Blunt vector. To avoid PCR-derived errors, more than four independent clones were sequenced. Signal peptide cleavage sites were predicted using the SignalP 4.1 Server^44^. Glycosylation sites were predicted using the NetNGlyc 1.0 Server (http://www.cbs.dtu.dk/services/NetNGlyc/) and the NetOGlyc 4.0 Server (http://www.cbs.dtu.dk/services/NetOGlyc/). The oligonucleotide primers used in this study are shown in Table S2. Sequence data have been submitted to the DDBJ/EMBL/GenBank databases under the accession numbers LC314430 –LC314439.

### Phylogenetic analysis

A phylogenetic analysis was performed for the millipede HNLs. Multiple sequence alignment of HNLs was performed using ClustalX^45^ with default parameters. The phylogenetic tree was constructed by the neighbor-joining method using MEGA 6 software^46^. The significance level for the phylogenetic tree was assessed by bootstrap testing with 1000 replications.

### Production of recombinant HNLs in insect cells

cDNAs encoding HNLs from millipedes were expressed as non-tagged proteins in Sf9 cells. Coding sequences of HNLs were re-amplified by PCR and inserted into the *Bam*HI–*Hin*dIII site of the pFastbac1 (Thermo Fisher Scientific) using the In-Fusion HD Cloning kit (Clontech Laboratories). After confirmation of the inserted DNA sequences, the plasmids were used to transform *E. coli* DH10BAC (Thermo Fisher Scientific) to obtain recombinant bacmids. Recombinant viruses were prepared as described previously^47^. Briefly, Sf9 cells maintained in Sf-900II-SFM medium (Thermo Fisher Scientific) containing 5% fetal bovine serum (FBS; Gibco BRL, Grand Island, NY, USA) at 28 °C were transfected with recombinant bacmids using X-tremeGENE 9 DNA Transfection Reagent (Roche, Basel, Switzerland). Recombinant viruses were amplified and P2 viruses were used to infect Sf9 cells; the virus preparation was stored at 4 °C. The titer of virus stocks was determined by quantitative real time PCR using *ie*-1-specific primers^48^. Viral genomic DNA was extracted from P2 virus stocks using the NucleoSpin Virus kit (Takara). Real-time PCR was performed using an ABI 7500 Real-Time PCR System (Applied Biosystems, Foster City, CA, USA) and SYBR premix Ex Taq II (Ti RNaseH Plus; Takara) with ROX as the reference dye. The PCR amplification conditions were as follows: 95 °C for 30 s, and 40 cycles at 95 °C for 5 s and at 60° for 40 s. A melting curve analysis was performed after the assay to verify specificity. The titer of samples was calculated from the standard curve generated from serial dilutions of a sample with known titer.

To produce HNLs, Sf9 cells (1.0 × 10^6^ cells/ml) maintained in Sf-900II-SFM supplemented with 5% FBS were infected with P2 viruses at multiplicity of infection of 1. Cells were removed by centrifugation (500 *g*, 4 °C, 5 min) at 72 h after infection and the culture supernatants were recovered.

### Production of recombinant HNLs in *E. coli*

cDNAs encoding HNLs from millipedes were expressed in *E. coli* BL21(DE3) or *E. coli* SHuffle T7 (New England BioLabs). SHuffle T7 constitutively expresses a chromosomal copy of disulfide-bond isomerase to promote the correction of mis-oxidized proteins^20^. To express HNLs in the cytosol of *E. coli*, we re-amplified HNLs without the signal peptide coding sequences and cloned each one into the *Nde*I–*Hin*dIII site of pET28 for expression as an N-terminal His-tagged protein. After confirmation of the inserted DNA sequences, the resultant plasmids were used to transform *E. coli* BL21(DE3) or SHuffle T7. Transformants were inoculated into LB medium containing kanamycin (50 µg/mL) and glucose (1% w/v), and cultured at 30 °C overnight. Cultures were transferred to 3 L of TB-based autoinduction medium and cultured at 26 °C for 24 h. Then, the cells were harvested by centrifugation to yield approximately 130 g of wet cells and resuspended in 300 mL of 10 mM HEPES-NaOH (pH 8.0) containing 0.1 M NaCl and 20 mM imidazole. After disrupting cells, insoluble materials were removed by centrifugation and the supernatant was collected as the cell-free extract. The extract was loaded onto a HisTrap HP column (GE Healthcare) equilibrated with 10 mM HEPES-NaOH (pH 8.0) containing 0.1 M NaCl and 20 mM imidazole, and then HNL was eluted with a linear gradient of imidazole (20–300 mM in the same buffer). The active fractions were pooled and loaded onto a Resource Q column (GE Healthcare) equilibrated with 10 mM HEPES-NaOH (pH 8.0), and eluted with a linear gradient of sodium chloride (0–300 mM in the same buffer). Protein concentrations were determined using the TaKaRa BCA Protein Assay Kit (Takara) with bovine serum albumin as the standard.

### Effect of temperature and pH on the recombinant Pton3HNL

The optimum temperature was evaluated by monitoring the (*R*)-mandelonitrile synthetic activity for 5 min at various temperature ranges from 15 °C to 55 °C. The optimum pH was determined by measuring the activity for 5 min in 300 mM sodium citrate buffer (pH 2.5–5.5). The thermostability was evaluated by pre-incubating the Pton3HNL at different temperatures (15 °C to 95 °C) in 10 mM KPB (pH 7.0) for 1 h, and then the residual enzyme activity was determined. The pH stability was determined by measuring the remaining activity after incubation for 1 h in buffers of varied pH (3.0–10.5). The buffers used were sodium citrate buffer (pH 3.0 to 6.0, 100 mM), KPB (pH 6.0 to 8.0, 100 mM), Tricine-NaOH buffer (pH 8.0–9.0, 100 mM), and sodium carbonate buffer (pH 9.0 to 10.5, 100 mM).

### CD spectroscopy analyses

The recombinant Pton3HNL used for CD measurements had protein concentration of 0.15 mg/mL. Samples were incubated in various buffers (3.0–10.0) at 25 °C or in KPB (pH 7.0) at various temperatures (25 °C–100 °C) for 1 h. Far-UV CD spectra were collected on a JASCO J-720W circular dichroism spectropolarimeter using 2-mm quartz cuvette (Tosoh) containing 500 µL of protein solution under the following condition; temperature, 20 °C; cell length, 0.02 cm; resolution, 0.2 nm; bandwidth, 2.0 nm; sensitivity, 100 mdeg; response, 1 s; speed, 100 nm.min^−1^; and accumulation, 10–20. Every sample was measured in triplicate and data were analyzed using the DichroWeb^49^ with the K2D method^50^.

### Production of (*R*)-mandelonitrile by whole recombinant *E. coli* cells

*E. coli* SHuffle cells expressing Pton3HNL were used as whole-cell biocatalysts for the synthesis of chiral cyanohydrins in aqueous buffer. After the culture as described above, cells were harvested from 0.8 mL culture to yield 35.2 ± 3.4 mg of wet cells and resuspended in 150 µl 0.4 M citrate buffer (pH 3.0). After adding 10 µl 1 M benzaldehyde and 20 µl 1 M KCN, the mixture was incubated at 22 °C for 5 min. Reaction products were extracted and analyzed by HPLC as described above. Enantiomeric excess was determined as described in a previous study^43^.

## Electronic supplementary material

Supplementary information

## References

[1] Zagrobelny M, Bak S, Møller BL (2008). Cyanogenesis in plants and arthropods. Phytochemistry.

[2] Dadashipour M, Asano Y (2011). Hydroxynitrile lyases: insights into biochemistry, discovery, and engineering. ACS Catal..

[3] Purkarthofer T, Skranc W, Schuster C, Griengl H (2007). Potential and capabilities of hydroxynitrile lyases as biocatalysts in the chemical industry. Appl. Microbiol. Biotechnol..

[4] Gleadow RM, Møller BL (2014). Cyanogenic glycosides: synthesis, physiology, and phenotypic plasticity. Annu. Rev. Plant Biol..

[5] Asano Y (2005). Screening for new hydroxynitrilases from plants. Biosci., Biotechnol., Biochem..

[6] Caruso CS (2009). α-Hydroxynitrile lyase protein from *Xylella fastidiosa*: cloning, expression, and characterization. Microb. Pathog..

[7] Hussain Z (2012). Characterization of two bacterial hydroxynitrile lyases with high similarity to cupin superfamily proteins. Appl. Environ. Microbiol..

[8] Hajnal I (2013). Biochemical and structural characterization of a novel bacterial manganese-dependent hydroxynitrile lyase. FEBS J..

[9] Nakano S, Dadashipour M, Asano Y (2014). Structural and functional analysis of hydroxynitrile lyase from *Baliospermum montanum* with crystal structure, molecular dynamics and enzyme kinetics. Biochim. Biophys. Acta.

[10] Dreveny I, Gruber K, Glieder A, Thompson A, Kratky C (2001). The hydroxynitrile lyase from almond: a lyase that looks like an oxidoreductase. Structure.

[11] Trummler K, Wajant H (1997). Molecular cloning of acetone cyanohydrin lyase from flax (*Linum usitatissimum*). Definition of a novel class of hydroxynitrile lyases. J. Biol. Chem..

[12] Lauble H, Miehlich B, Förster S, Wajant H (2002). Crystal structure of hydroxynitrile lyase from *Sorghum bicolor* in complex with the inhibitor benzoic acid: A novel cyanogenic enzyme. Biochemistry.

[13] Bracco P, Busch H, Langermann, von J, Hanefeld U (2016). Enantioselective synthesis of cyanohydrins catalysed by hydroxynitrile lyases - a review. Org. Biomol. Chem..

[14] Brewer MS, Sierwald P, Bond JE (2012). Millipede taxonomy after 250 years: Classification and taxonomic practices in a mega-diverse yet understudied arthropod group. PLoS ONE.

[15] Shear WA (2015). The chemical defenses of millipedes (diplopoda): biochemistry, physiology and ecology. Biochem. Syst. Ecol..

[16] Eisner T, Eisner HE, Hurst JJ, Kafatos FC, Meinwald J (1963). Cyanogenic glandular apparatus of a millipede. Science.

[17] Meyer-Rochow VB (2015). New observations - with older ones reviewed - on mass migrations in millipedes based on a recent outbreak on Hachijojima (Izu Islands) of the polydesmid diplopod (*Chamberlinius hualienensis*, Wang 1956): nothing appears to make much sense. Zool. Res..

[18] Dadashipour M, Ishida Y, Yamamoto K, Asano Y (2015). Discovery and molecular and biocatalytic properties of hydroxynitrile lyase from an invasive millipede, *Chamberlinius hualienensis*. Proc. Natl. Acad. Sci. USA.

[19] Glieder A (2003). Comprehensive step-by-step engineering of an (*R*)-hydroxynitrile lyase for large-scale asymmetric synthesis. Angew. Chem. Int. Ed. Engl..

[20] Lobstein J (2012). SHuffle, a novel *Escherichia coli* protein expression strain capable of correctly folding disulfide bonded proteins in its cytoplasm. Microb. Cell Fact..

[21] Anton BP, Fomenkov A, Raleigh EA, Berkmen M (2016). Complete genome sequence of the engineered *Escherichia coli* SHuffle strains and their wild-type parents. Genome Announc.

[22] Wachtmeister J, Rother D (2016). Recent advances in whole cell biocatalysis techniques bridging from investigative to industrial scale. Curr. Opin. Biotechnol..

[23] Nanda S, Kato Y, Asano Y (2005). A new (*R*)-hydroxynitrile lyase from *Prunus mume*: asymmetric synthesis of cyanohydrins. Tetrahedron.

[24] Yin J, Li G, Ren X, Herrler G (2007). Select what you need: a comparative evaluation of the advantages and limitations of frequently used expression systems for foreign genes. J. Biotechnol..

[25] Zhao G-J, Yang Z-Q, Guo Y-H (2011). Cloning and expression of hydroxynitrile lyase gene from *Eriobotrya japonica* in *Pichia pastoris*. J. Biosci. Bioeng..

[26] Fukuta Y (2011). Characterization of a new (*R*)-hydroxynitrile lyase from the Japanese apricot *Prunus mume* and cDNA cloning and secretory expression of one of the isozymes in *Pichia pastoris*. Biosci., Biotechnol., Biochem..

[27] Weis R, Gaisberger R, Gruber K, Glieder A (2007). Serine scanning: a tool to prove the consequences of *N*-glycosylation of proteins. J. Biotechnol..

[28] Hasslacher M (1997). High-level intracellular expression of hydroxynitrile lyase from the tropical rubber tree *Hevea brasiliensis* in microbial hosts. Protein Expr. Purif..

[29] Inan M, Aryasomayajula D, Sinha J, Meagher MM (2006). Enhancement of protein secretion in *Pichia pastoris* by overexpression of protein disulfide isomerase. Biotechnol. Bioeng..

[30] Gasser B, Maurer M, Gach J, Kunert R, Mattanovich D (2006). Engineering of *Pichia pastoris* for improved production of antibody fragments. Biotechnol. Bioeng..

[31] Nuylert A, Ishida Y, Asano Y (2017). Effect of glycosylation on the biocatalytic properties of hydroxynitrile lyase from the passion fruit, *Passiflora edulis*: a comparison of natural and recombinant enzymes. Chembiochem.

[32] Dombkowski AA, Sultana KZ, Craig DB (2014). Protein disulfide engineering. FEBS Lett..

[33] Ueatrongchit T, Tamura K, Ohmiya T, H-Kittikun A, Asano Y (2010). Hydroxynitrile lyase from *Passiflora edulis*: purification, characteristics and application in asymmetric synthesis of (*R*)-mandelonitrile. Enzyme Microb. Technol..

[34] Bauer M, Griengl H, Steiner W (1999). Parameters influencing stability and activity of a *S*-hydroxynitrile lyase from *Hevea brasiliensis* in two-phase systems. Enzyme Microb. Technol..

[35] Hanefeld U (2013). Immobilisation of hydroxynitrile lyases. Chem. Soc. Rev..

[36] Ishige T, Honda K, Shimizu S (2005). Whole organism biocatalysis. Curr. Opin. Chem. Biol..

[37] Yamamoto K, Asano Y (2015). Efficient production of lumichrome by *Microbacterium* sp. strain TPU 3598. Appl. Environ. Microbiol..

[38] Scholz KE (2012). Synthesis of chiral cyanohydrins by recombinant *Escherichia coli* cells in a micro-aqueous reaction system. Appl. Environ. Microbiol..

[39] Sheldon RA (2005). Green solvents for sustainable organic synthesis: state of the art. Green Chem..

[40] Yamaguchi T, Kuwahara Y, Asano Y (2017). A novel cytochrome P450, CYP3201B1, is involved in (*R*)‐mandelonitrile biosynthesis in a cyanogenic millipede. FEBS Open Bio.

[41] Sota T, Tanabe T (2010). Multiple speciation events in an arthropod with divergent evolution in sexual morphology. Proc. Biol. Sci..

[42] Tanabe T (2010). Revision of the millipede genus *Parafontaria* Verhoeff, 1936 (Diplopoda, Xystodesmidae). J. Nat. Hist..

[43] Ishida Y (2016). A sacrificial millipede altruistically protects its swarm using a drone blood enzyme, mandelonitrile oxidase. Sci. Rep..

[44] Petersen TN, Brunak S, Heijne, von G, Nielsen H (2011). SignalP 4.0: discriminating signal peptides from transmembrane regions. Nat. Methods.

[45] Larkin MA (2007). Clustal W and Clustal X version 2.0. Bioinformatics.

[46] Tamura K, Stecher G, Peterson D, Filipski A, Kumar S (2013). MEGA6: Molecular evolutionary genetics analysis version 6.0. Mol. Biol. Evol..

[47] Yamaguchi T, Noge K, Asano Y (2016). Cytochrome P450 CYP71AT96 catalyses the final step of herbivore-induced phenylacetonitrile biosynthesis in the giant knotweed, *Fallopia sachalinensis*. Plant Mol. Biol..

[48] Lo H-R, Chao Y-C (2004). Rapid titer determination of baculovirus by quantitative real-time polymerase chain reaction. Biotechnol. Prog..

[49] Whitmore L, Wallace BA (2004). DICHROWEB, an online server for protein secondary structure analyses from circular dichroism spectroscopic data. Nucleic Acids Res..

[50] Andrade MA, Chacón P, Merelo JJ, Morán F (1993). Evaluation of secondary structure of proteins from UV circular dichroism spectra using an unsupervised learning neural network. Protein Eng..

