# Supplementary material for: Hydroxynitrile lyases from cyanogenic millipedes: molecular cloning, heterologous expression, and whole-cell biocatalysis for the production of (R)-mandelonitrile
Source: Sci Rep. 2018 Feb 14;8:3051. doi: 10.1038/s41598-018-20190-x (PMC5813103; doi:10.1038/s41598-018-20190-x)
Supplement: Supplementary file 1 — Supplementary information [file 41598_2018_20190_MOESM1_ESM.pdf]

Hydroxynitrile lyases from cyanogenic millipedes: molecular cloning, heterologous expression, and whole-cell biocatalysis for the production of (*R*)-mandelonitrile

Takuya Yamaguchi[1,2], Aem Nuylert[1,2], Atsutoshi Ina[1,2], Tsutomu Tanabe[3], and Yasuhisa Asano[1,2]

Figure legends

Fig. S1. A swarm of *Nedyopus tambanus tambanus*.

Fig. S2. Far-UV circular dichroism (CD) spectra of the recombinant Pton3HNL after incubation in various pH (a) or temperatures (b)

Fig. S3. Synthesis of (*R*)-madenlonitrile from benzaldehyde and KCN using the whole recombinant *E. coli* cells expressing Pton3HNL under the optimized condition.
Benzaldehyde and mandelonitrile were measured by monitoring the absorbance at 254 nm using an HPLC equipped with a chiral column, and the inset shows a magnified view of the chromatogram for a retention time between 10 min and 15 min. Retention times of benzaldehyde, (*R*)-mandelonitrile, and (*S*)-mandelonitrile were 5.5 min, 11 min and 13.8 min, respectively.

Fig. S4. The full-length gel image associated to figure 1.

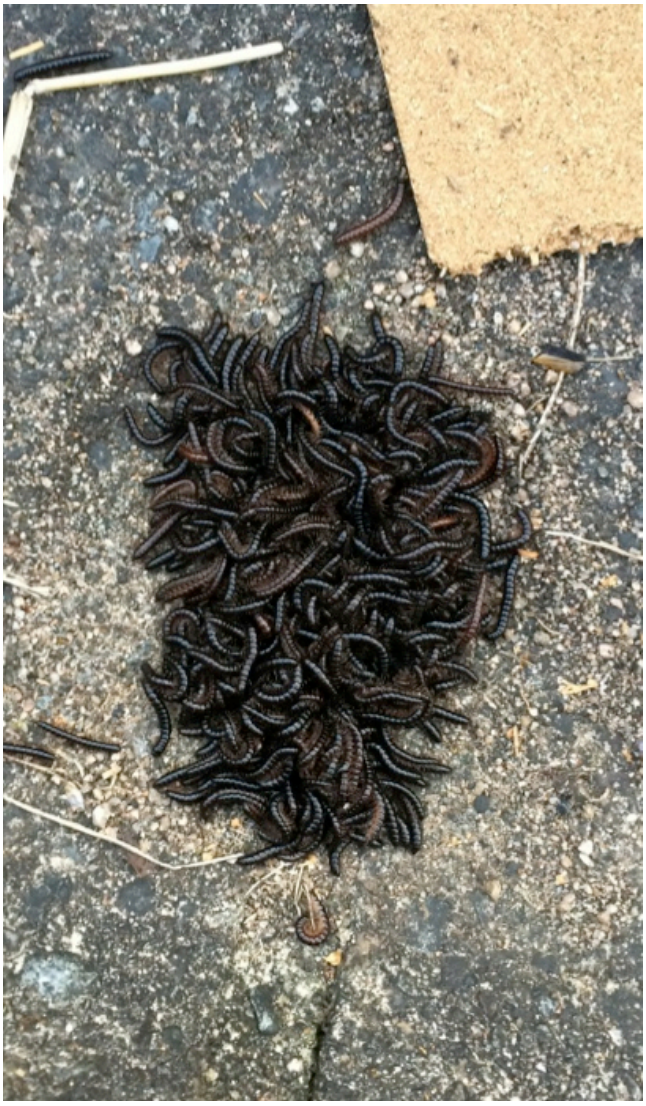

Fig. S1. A swarm of *Nedyopus tambanus tambanus.*

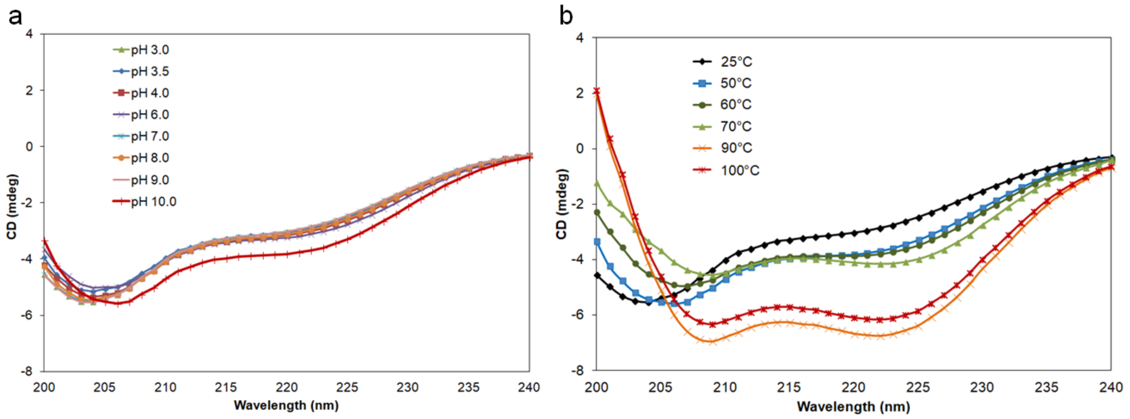

Fig. S2. Far-UV circular dichroism (CD) spectra of the recombinant Pton3HNL after incubation in various pH (a) or temperatures (b)

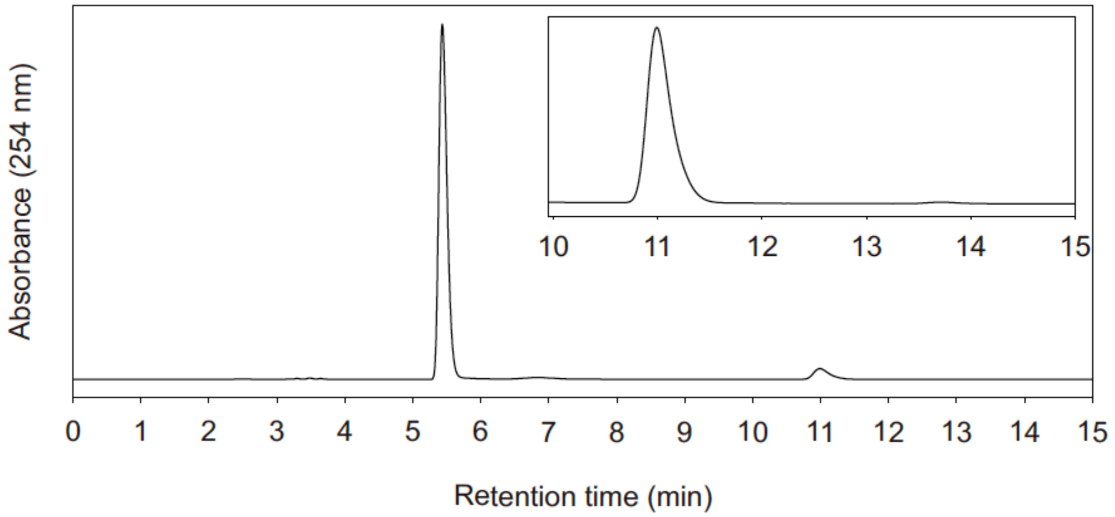

Fig. S3. Synthesis of (*R*)-madenlonitrile from benzaldehyde and KCN using the whole recombinant *E. coli* cells expressing Pton3HNL under the optimized condition. Benzaldehyde and mandelonitrile were measured by monitoring the absorbance at 254 nm using an HPLC equipped with a chiral column, and the inset shows a magnified view of the chromatogram for a retention time between 10 min and 15 min. Retention times of benzaldehyde, (*R*)-mandelonitrile, and (*S*)-mandelonitrile were 5.5 min, 11 min and 13.8 min, respectively.

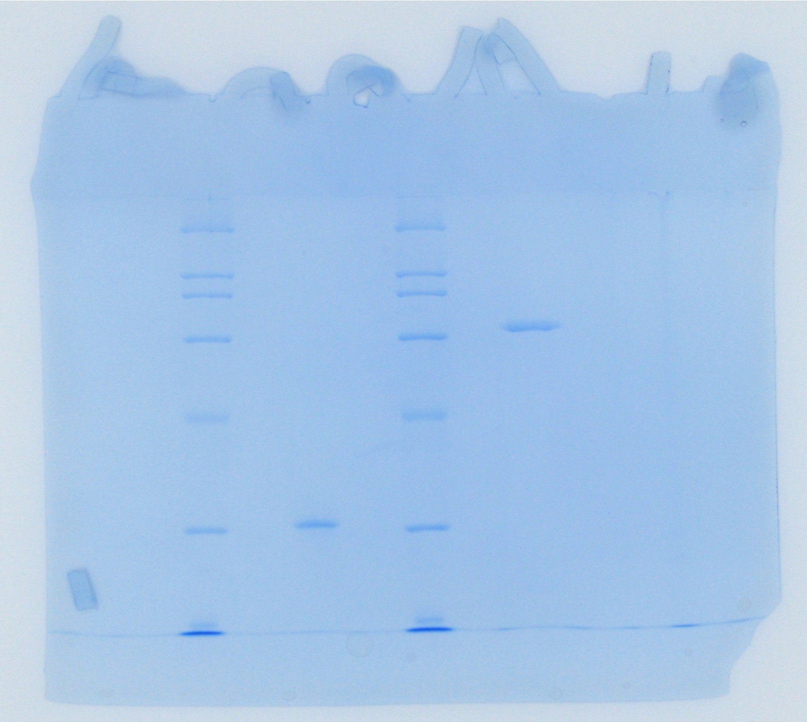

Fig. S4. The full-length gel image associated to figure 1.

Table S1. Putative signal peptides and glycosylation sites of millipede HNLs

	Signal peptide amino acid sequence (amino acid position in protein)	*N*-linked glycosylation site	*O*-linked glycosylation site
NttHNL	MLFYVSILLVVTLTGAEE (1-16)	N^{99}, N^{119}, N^{129}, N^{143}	
NtmHNL	MLFYVSILLVVALAGA (1-16)	N^{92}, N^{99}, N^{143}	
OgraHNL	MLYYVSILLMAVYAVAVA (1-18)	N^{94}, N^{101}, N^{121}, N^{145}	
PfalHNL	MTSIIFLTTVALIVMLAELGWA (1-22)	N^{75}	
PtokHNL	MTSIILLTTVALSVMLAELGWA (1-22)	N^{74}	
Pton1HNL	MTSIILLLAAALTVMLAELGWA (1-22)	N^{75}	
Pton2HNL	MTSIILLTTVALIVMLAELGWA (1-22)	N^{74}	
Pton3HNL	MTSIILLTTVALIVMLAELGWA (1-22)	N^{75}, N^{136}	S^{36}, S^{165}
RssHNL	MTSIMLCLTLALTAMMAELGCA (1-22)	N^{76}	
RspHNL	MTSIMFSLTLALTAMMAELGWA (1-22)	N^{76}	

Table S2. Oligonucleotide primers

Primers	Sequences (5’-3’)
For DOP-PCR	
NttHNL-DOPF1	GARGARGARCCNCTAAC
NttHNL-DOPF2	GARGARGARCCNCTTAC
NttHNL-DOPF3	GARGARGARCCNCTGAC
NttHNL-DOPF4	GARGARGARCCNCTCAC
NttHNL-DOPR1	GTNAGNGGYTCYTCTTC
NttHNL-DOPR2	GTNAGNGGYTCYTCCTC
HNL-FWver1	CTGCAACTGCATTGGAMATTCAAGG
HNL-RVver1	ATGAATCTTRTCRCCGTTTGGAAC
HNL-FWver2	SSAACTGCATTGGAYATMMRAGG
HNL-RVver2	ATGAATCTTRTCRCCRTTTGGRAC
For 5’- and 3’- RACE	
NttHNL-F1	GTTCCAGTTCCTCCGTTAGAAGATTTT
NttHNL-F2	CCCAGGCTGCAACTGCATTGGACATT
NttHNL-R1	CTCTGCAATTGCAGAACCATTGCACGTA
NttHNL-R2	CCATTTGGGGTGTTCAAATTAGTATATT
NtmHNL-F1	GTTCCAGTTCCTCCGTTAGAAGATTTT
NtmHNL-F2	CCCAGGCTGCAACTGCATTGGACATT
NtmHNL-R1	CTCTGCAATTGCAGAACCATTGCACGTA
NtmHNL-R2	CCATTTGGGGTGTTCAAATTAGTATATT
OgraHNL-F1	CGTTGGTGGTCCTAATAATTCAGCTAT
OgraHNL-F2	CACCAATCTGAACACTCCAAATGGAA
OgraHNL-R1	GGATCGGTTCCGTTGTTGTTATTA
OgraHNL-R2	CGTATAGGCGCAATAGGAGCTTCCATT
PfalHNL-F1	GACTTCACCATTGGTTCTGATTCTAT
PfalHNL-F2	CCCCAAGGTGCCAACTATTGTGCATA
PfalHNL-R1	CGGGACCATTGCAAGAGTATGCACAA
PfalHNL-R2	GGGGATCATTTAGGTCGATATATTGAA
PtokHNL-F1	GGACAGCCTTTTCGACTAATTGTGAT

PtokHNL-F2	CCCAAGGTGCCAACTACTGTGCATA
PtokHNL-R1	GCCTGGAGTTGTTGAGGCGATATGTAT
PtokHNL-R2	GCAAGAGTAGCCTATGCACAGTAGTTG
PtonHNL-F1	GGTCCCGATGCTATGACGGCCTATTT
PtonHNL-F2	GGTGCCAACTATTGTGCATACTTTT
PtonHNL-R1	GCCTGGAGTTGTTGAGGCGATATGTA
PtonHNL-R2	GGGACCATTGCAAAAGTATGCACAATA
Pton2HNL-F1	CCGATGGTCTGACAGCCTATTTGACTA
Pton2HNL-F2	CCCCAAGGTGCCAACTACTGTGCATA
Pton2HNL-R1	GGCGATATGTATCCAGTATTCGTAGTGCA
Pton2HNL-R2	CGGGACCATTGCAAGAGTATGCACAGT
Pton3HNL-F1	CTGACAGCCTATTTGACTAATTGTGAT
Pton3HNL-F2	GCATACTCTTGCAATGGTTCCGAAA
Pton3HNL-R1	GCCTGGAGTTGTTGAGGCGATATGTAT
Pton3HNL-R2	CGGAACCATTGCAAGAGTATGCACA
RssHNL-F1	GACTTCCTCATCGCTCCTGATTGTAT
RssHNL-F2	CGTCGAGGATCCCAAGGGTGCCAA
RssHNL-R1	GCCAGCTATATTGGAAGTGCATTT
RssHNL-R2	CCATCGCAAGAGTATGCGCAATAGTT
RspHNL-F1	CCGGGGCAAAACAGGTTTGGTA
RspHNL-F2	GGGTGCCAACTATTGCGCATACTCTT
RspHNL-R1	GCCAGTATTGGAAGTGCATTTGTATT
RspHNL-R2	CAGGGGATCATCGAGGTCGACATATT
For amplification of HNL	
ChuaHNL-FW	GGATCCATGTTGAGTTCACTAGTAGTAACAGTAA
ChuaHNL-RV	AAGCTTAGTAAAAAGCAAAGCAACCGTGGGTTTCG
NttHNL-FW	ATGCTGTTTTACGTTTCGATTCTTCTAG
NttHNL-RV	TTAATAGAAAGCAAAACAACCATGGTG
NtmHNL-FW	ATGCTGTTTTACGTCTCGATTCTTC
NtmHNL-RV	TCAATAGAAAGCAAAACAGCCATGG
OgraHNL-FW	ATGTTGTACTACGTTTCAATACTTT

OgraHNL-RV	CTAATAGAAAGCAAAACAGCCATGG
PfalHNL-FW	ATGACTTCGATCATTTTCCTCACG
PfalHNL-RV	TTAGTAATAGAGAGGACAGAAAGGG
PtokHNL-FW	ATGACTTCGATCATTCTCCTCACG
PtokHNL-RV	TTAGTAATAGAGGGGACAGAAAAGG
PtonHNL-FW	ATGACTTCAATCATTCTCCTCTTGG
PtonHNL-RV	TTAGTAATAGAGAGGACAGAAAGGGTG
Pton2HNL-FW	ATGACTTCGATCATTCTCCTCACG
Pton2HNL-RV	TTAGTAATAGAGAGGACAGTAAAGGTG
Pton3HNL-FW	ATGACTTCGATCATTCTCCTCACG
Pton3HNL-RV	TTAGTAATAGAGAGGACAGTAAAGG
RssHNL-FW	ATGACTTCGATCATGCTCTGTTTAAC
RssHNL-RV	TTAGCTATAGAAGGGGCAGAAAGGG
RspHNL-FW	ATGACTTCGATCATGTTCAGCCTG
RspHNL-RV	TTAGCTATAGAAGGGGCAGATAGGG

For preparing insert DNA into pFastbac1 expression vector

IFSf-ChuaHNL-FW	CATCGGGCGCGGATCCATGTTGAGTTCACTAGTAG
IFSf-ChuaHNL-RV	ACTTCTCGACAAGCTTAGTAAAAAGCAAAGCAACCGTG
IFSf-NttHNL-FW	CATCGGGCGCGGATCCATGCTGTTTTACGTTTCGATTC
IFSf-NttHNL-RV	ACTTCTCGACAAGCTTTTAATAGAAAGCAAAACAACCATGG
IFSf-NtmHNL-FW	CATCGGGCGCGGATCCATGCTGTTTTACGTTTCGATTCTTC
IFSf-NtmHNL-RV	ACTTCTCGACAAGCTTTCAATAGAAAGCAAAACAGCCATGG
IFSf-OgraHNL-FW	CATCGGGCGCGGATCCATGTTGTACTACGTTTCAATAC
IFSf-OgraHNL-RV	ACTTCTCGACAAGCTTCTAATAGAAAGCAAAACAGCCATG
IFSf-PfalHNL-FW	CATCGGGCGCGGATCCATGACTTCGATCATTTTCCTCACG
IFSf-PfalHNL-RV	ACTTCTCGACAAGCTTTTAGTAATAGAGAGGACAGAAAGGG
IFSf-PtokHNL-FW	CATCGGGCGCGGATCCATGACTTCGATCATTCTCCTCACG
IFSf-PtokHNL-RV	ACTTCTCGACAAGCTTTTAGTAATAGAGGGGACAGAAAAGG
IFSf-PtonHNL-FW	CATCGGGCGCGGATCCATGACTTCAATCATTCTCCTCTTG
IFSf-PtonHNL-RV	ACTTCTCGACAAGCTTTTAGTAATAGAGAGGACAGAAAGGGTG
IFSf-Pton2HNL-FW	CATCGGGCGCGGATCCATGACTTCGATCATTCTCCTCACG

IFSf-Pton2HNL-RV	ACTTCTCGACAAGCTTTTAGTAATAGAGAGGACAGTAAAGGTG
IFSf-Pton3HNL-FW	CATCGGGCGCGGATCCATGACTTCGATCATTCTCCTCACG
IFSf-Pton3HNL-RV	ACTTCTCGACAAGCTTTTAGTAATAGAGAGGACAGTAAAGG
IFSf-RssHNL-FW	CATCGGGCGCGGATCCATGACTTCGATCATGCTCTGTTTA
IFSf-RssHNL-RV	ACTTCTCGACAAGCTTTTAGCTATAGAAGGGGCAGAAAGGG
IFSf-RspHNL-FW	CATCGGGCGCGGATCCATGACTTCGATCATGTTCAGCCTG
IFSf-RspHNL-RV	ACTTCTCGACAAGCTTTTAGCTATAGAAGGGGCAGATAGGG
For preparing insert DNA into pET28	
IFEc-ChuaHNL-FW	GCCGCGCGGCAGCCATATGCTGACTTGTGATCAACTTCCCAA
IFEc-ChuaHNL-RV	GTGCGGCCGCAAGCTTAGTAAAAAGCAAAGCAACCGTGGG
IFEc-NttHNL-FW	GCCGCGCGGCAGCCATATGGAGGAGGAACCATTGACATGCG
IFEc-NttHNL-RV	GTGCGGCCGCAAGCTTTTAATAGAAAGCAAAACAACCATGG
IFEc-NtmHNL-FW	GCCGCGCGGCAGCCATATGGAGGACGAACCATTGACTTGCGATA
IFEc-NtmHNL-RV	GTGCGGCCGCAAGCTTTCAATAGAAAGCAAAACAGCCATGG
IFEc-OgraHNL-FW	GCCGCGCGGCAGCCATATGGATGAAGACCCAATGACTTGCG
IFEc-OgraHNL-RV	GTGCGGCCGCAAGCTTCTAATAGAAAGCAAAACAGCCATGG
IFEc-PfalHNL-FW	GCCGCGCGGCAGCCATATGCAACCTTCAGGTCTCACTTGCGACC
IFEc-PfalHNL-RV	GTGCGGCCGCAAGCTTAGTAATAGAGAGGACAGAAAGGG
IFEc-PtokHNL-FW	GCCGCGCGGCAGCCATATGGTGTCAGCATTGACTTGCGACCATC
IFEc-PtokHNL-RV	GTGCGGCCGCAAGCTTTTAGTAATAGAGGGGACAGAAAAGG
IFEc-PtonHNL-FW	GCCGCGCGGCAGCCATATGCAACCTTCAGGTCTCGCTTGCGAC
IFEc-PtonHNL-RV	GTGCGGCCGCAAGCTTTTAGTAATAGAGAGGACAGAAAGGG
IFEc-Pton2HNL-FW	GCCGCGCGGCAGCCATATGGTGTCAGGTTTGACTTGCGACCAGC
IFEc-Pton2HNL-RV	GTGCGGCCGCAAGCTTTTAGTAATAGAGAGGACAGTAAAGGTG
IFEc-Pton3HNL-FW	GCCGCGCGGCAGCCATATGCAACCTTCAGGTCTCACTTGCGAC
IFEc-Pton3HNL-RV	GTGCGGCCGCAAGCTTTTAGTAATAGAGAGGACAGTAAAGG
IFEc-RssHNL-FW	GCCGCGCGGCAGCCATATGCAACCGTCTGAAGGCCCTTCCTGC
IFEc-RssHNL-RV	GTGCGGCCGCAAGCTTTTAGCTATAGAAGGGGCAGAAAGGG
IFEc-RspHNL-FW	GCCGCGCGGCAGCCATATGCAACCTCCTGACGGCCCTTCCTGCG
IFEc-RspHNL-RV	GTGCGGCCGCAAGCTTAGCTATAGAAGGGGCAGATAGGG

77
